# Evaluating the Role of N-Acetyl-L-Tryptophan in the Aβ 1-42-Induced Neuroinflammation and Cognitive Decline in Alzheimer’s Disease

**DOI:** 10.1007/s12035-023-03844-4

**Published:** 2023-12-13

**Authors:** Sairaj Satarker, Prasada Chowdari Gurram, Ajmal Nassar, Suman Manandhar, RJA Vibhavari, Dani Lakshman Yarlagadda, Jayesh Mudgal, Shaila Lewis, Devinder Arora, Madhavan Nampoothiri

**Affiliations:** 1 Department of Pharmacology, Manipal College of Pharmaceutical Sciences, Manipal Academy of Higher Education, Manipal, Karnataka 576104 India; 2 Department of Pharmaceutical Quality Assurance, Manipal College of Pharmaceutical Sciences, Manipal Academy of Higher Education, Manipal, Karnataka 576104 India; 3 Department of Pharmaceutics, Manipal College of Pharmaceutical Sciences, Manipal Academy of Higher Education, Manipal, Karnataka 576104 India; 4 School of Pharmacy and Medical Sciences, Griffith University, QLD, Gold Coast, 4222 Australia

**Keywords:** N-Acetyl-L-tryptophan, Alzheimer’s disease, Neuroinflammation, Substance P, Aβ oligomers

## Abstract

Alzheimer’s disease (AD), a neurodegenerative condition previously known to affect the older population, is also now seen in younger individuals. AD is often associated with cognitive decline and neuroinflammation elevation primarily due to amyloid β (Aβ) accumulation. Multiple pathological complications in AD call for therapies with a wide range of neuroprotection. Our study aims to evaluate the effect of N-acetyl-L-tryptophan (NAT) in ameliorating the cognitive decline and neuroinflammation induced by Aβ 1-42 oligomers and to determine the therapeutic concentration of NAT in the brain. We administered Aβ 1-42 oligomers in rats via intracerebroventricular (i.c.v.) injection to induce AD-like conditions. The NAT-treated animals lowered the cognitive decline in the Morris water maze characterized by shorter escape latency and increased path efficiency and platform entries. Interestingly, the hippocampus and frontal cortex showed downregulation of tumor necrosis factor, interleukin-6, and substance P levels. NAT treatment also reduced acetylcholinesterase activity and total and phosphorylated nuclear factor kappa B and Tau levels. Lastly, we observed upregulation of cAMP response element-binding protein 1 (CREB1) signaling. Surprisingly, our HPLC method was not sensitive enough to detect the therapeutic levels of NAT in the brain, possibly due to NAT concentrations being below the lowest limit of quantification of our validated method. To summarize, the administration of NAT significantly lowered cognitive decline, neuroinflammatory pathways, and Tau protein and triggered the upregulation of CREB1 signaling, suggesting its neuroprotective role in AD-like conditions.

## Introduction

In normal physiology, the central nervous system (CNS) efficiently regulates cellular and molecular processes in the body. However, certain endogenous or exogenous stimuli arising due to multifaceted conditions in the CNS can disrupt its normal regulation owing to the development of complex pathophysiology, ultimately progressing to neurodegeneration. One such neurodegenerative diseases include Alzheimer’s disease (AD). AD is a highly prevalent type of dementia, affecting nearly 8.8 million individuals in India as of now [1]. Enormous efforts are made to understand the pathophysiology of AD. Among the several hypotheses involved in AD [2, 3], the Aβ cascade, Tau, cholinergic, and inflammation hypotheses grab our attention. The Aβ cascade hypothesis involves the mechanisms of β-secretase and γ-secretase-mediated formation and accumulation of Aβ from amyloid precursor protein (APP) [4], including the η-secretases as well [5]. The Tau hypothesis underpins the stages involved in the excessive phosphorylation of a microtubule-associated protein called Tau that destabilizes microtubules [6]. The cholinergic hypothesis focuses on the effects of reduced acetylcholine (ACh) neurotransmission in memory decline associated with AD conditions [7]. Finally, the inflammation hypothesis puts the reactive microglia, astrocytes, and the associated neuroinflammatory mechanisms in the center stage to describe their potential role in AD progression. Therefore, these hypotheses call for effective therapies having multiple targets to overcome such diverse effects of AD.

Considering these hypotheses, we aimed to explore the role of a tryptophan derivative, N-acetyl tryptophan (NAT), in the conditions representing AD, developed through intracerebroventricular (i.c.v.) injection of Aβ 1-42 in rats. The Aβ 1-42 is highly toxic to the CNS. This could be due to its structured C terminus and β-hairpin formed by the residues 31-34 and 38-41. As a result, the flexibility in the C terminus is lowered, thus enhancing its conversion into toxic oligomers [8]. Various solvents, incubation time, and temperatures influence the preparation of Aβ oligomers and fibrils [9–12]. The Aβ primarily exists as oligomers and fibrils, out of which the oligomeric Aβ assembled with a size range of 8–15 nm are the most toxic [13, 14]. These morphologies are mainly characterized using atomic force microscopy [15–17].

The i.c.v. administration of Aβ 1-42 in rodents is widely utilized in modeling AD [14, 18–21]. Interestingly, the Aβ affects cognition in the younger population much more than the elderly population, where the brain atrophy overweighs the cognitive decline effects of Aβ [22]. The regions of the hippocampus and frontal cortex are associated with cognitive abilities. AD conditions primarily influence spatial memory [23, 24]. *In vivo* evaluations employ the Morris water maze (MWM) to evaluate spatial memory in rodents [25–29]. To briefly put forth, diverse pathomechanisms could exert their actions on cognition in AD. High levels of nuclear factor kappa B (NFκB p65) and elevated BACE1 mediate Aβ production from APP [30, 31]. Impaired cAMP response element-binding protein (CREB) dysregulation alters cognition in AD [32]. In neuroblastoma cells, Aβ triggers acetylcholinesterase (AChE) activity [33]. The levels of phosphorylated Tau (p-Tau), especially p-Tau-Ser^396^, increased in rat hippocampal cultures [34] and human AD brains [35]. Profound upregulation in various neuroinflammatory markers like tumor necrosis factor α (TNF-α) and interleukin-6 (IL-6) is also evident in AD [36]. Another neuromodulator, substance P (SP), marks its presence in the area of neuroinflammation and cognition. In the CNS, the SP exerts a bidirectional role with neurotoxic and neuroprotective effects [37, 38], encouraging us to explore the role of NAT in the Aβ 1-42 rat model. Therefore, it is essential to understand these pathomechanisms collectively to aid better therapies for AD.

Therefore, therapies that target the multiple role players of AD can prove to be beneficial in AD therapeutics. One such peptide, NAT, has grabbed our attention. Very sparse literature exists on the neuroprotective actions of NAT in CNS. Previously, NAT has demonstrated neuroprotective roles in amyotrophic lateral sclerosis [37, 38], Parkinson’s disease [39], traumatic brain injury [40, 41], stroke [42], and radioprotection in CNS [43]. Previously, in our laboratory, NAT demonstrated neuroprotective effects on aluminum chloride-induced dementia in rats [44]. However, NAT has not been evaluated *in vivo* for its effects in AD-like conditions. Owing to the multiple neuroprotective effects of NAT in other neurodegenerative conditions mentioned previously, we suspect that NAT may also extrapolate similar effects in AD. This forms the rationale for choosing NAT in our study. Thus, we believe that, for the first time, we report the effect of NAT in the conditions of AD induced by i.c.v. administration of Aβ 1-42 oligomers in Wistar rats. Our study aimed to evaluate the actions of NAT on cognitive decline and neuroinflammation mediated by alterations in NFκB, CREB, Tau, and neuroinflammatory signaling in AD. We also attempted to investigate the levels of NAT in the rat brain, responsible for exerting neuroprotective actions in AD, using high-performance liquid chromatography (HPLC).

## Materials and Methods

### Animals

Male Wistar rats (4 months, 250–300 g) were used for the study. The Central Animal Research Facility (CARF) at Manipal Academy of Higher Education, India, provided the animals. The experimental techniques followed the guidelines of the Committee for Control and Supervision of Experiments on Animals (CCSEA). The experimental protocol bearing registration number IAEC/KMC/129/2019 was approved by the Institutional Animal Ethics Committee (IAEC). Two animals were housed per cage made of polypropylene. The animals were maintained at a temperature of 24 ± 2 °C with a relative humidity of 51% and a 12-h light/dark cycle. Food and water were provided *ad libitum*. One week before experimentation, the animals were handled to reduce experimenter-induced stress.

### Chemicals and Reagents

All the chemicals and reagents used in the study were of analytical research grade. Amyloid beta peptide, protease and phosphatase inhibitor cocktail, bicinchoninic acid (BCA) protein assay kit, and enhanced chemiluminescent substrate (ECL) were obtained from Thermo Fisher Scientific (MA, USA). N-Acetyl-L-tryptophan (NAT) and 1,1,1,3,3,3-hexafluoroisopropanol (HFIP) were purchased from Sigma-Aldrich (St. Louis, MO, USA). Phospho-nuclear factor kappa B p65 (P-NFκB p65), total NFκB (T-NFκB) p65, phospho-cyclic AMP-responsive element-binding protein 1 (P-CREB1), total CREB1 (T-CREB1), phospho-Tau (P-Tau), total Tau (T-Tau) primary antibodies, and goat anti-rabbit IgG(H+L)(peroxidase/HRP conjugated) secondary antibody were obtained from Elabscience (TX, USA). Precision plus protein dual color standards and sodium dodecyl sulfate were obtained from Bio-Rad (CA, USA). The Amersham Hybond poly(vinylidene fluoride) (PVDF) membrane was procured from General Electric Healthcare (UK). Bovine serum albumin (BSA) and anhydrous dimethyl sulfoxide (DMSO) were obtained from Sisco Research Laboratories (Mumbai, India). HPLC-grade methanol was obtained from Rankem, India.

### Preparation of Aβ 1-42 Oligomers

The Aβ 1-42 oligomers were prepared per previous literature with slight modifications [11, 12]. The Aβ 1-42 peptide (1 mg) was dissolved with 0.222 ml of HFIP to produce 1 mM Aβ-HFIP solution. HFIP was allowed to evaporate overnight. Then, 2 μl of anhydrous DMSO was added to dissolve the thin film of Aβ 1-42 obtained along the walls of the tubes. This 5-mM Aβ-DMSO solution was vortexed for 30 s and sonicated for 10 min in a bath sonicator. To this, 98 μl of cold 1× PBS pH 7.4 was added to produce a concentration of 100-μM Aβ1-42 solution. This solution was incubated at 4 °C for 72 h to facilitate oligomerization. Finally, a 30-μM Aβ1-42 oligomer solution was prepared and injected into the animals.

### Atomic Force Microscopy

The formation of Aβ 1-42 oligomers was imaged using Innova SPM Atomic Force Microscope (Bruker, USA) in tapping mode [17]. A 10-μl sample of 30 μM Aβ 1-42 was placed on a clean glass coverslip and air-dried at room temperature. A silicon probe (Tap300AI-G, Budget Sensors, Bulgaria) with a resonance frequency of 300 kHz and a force constant of 40 N/m was used. Images were developed with a scan rate of 1 Hz and a scan range of 5 μm × 5 μm. The raw images of the Aβ 1-42 oligomers were obtained using NanoScope analysis software (Bruker, USA). Further, image processing and oligomer size analysis were done using Gwyddion 2.63 software [45], as stated in the previous literature [46].

### Study Design

**Fig. 1:**
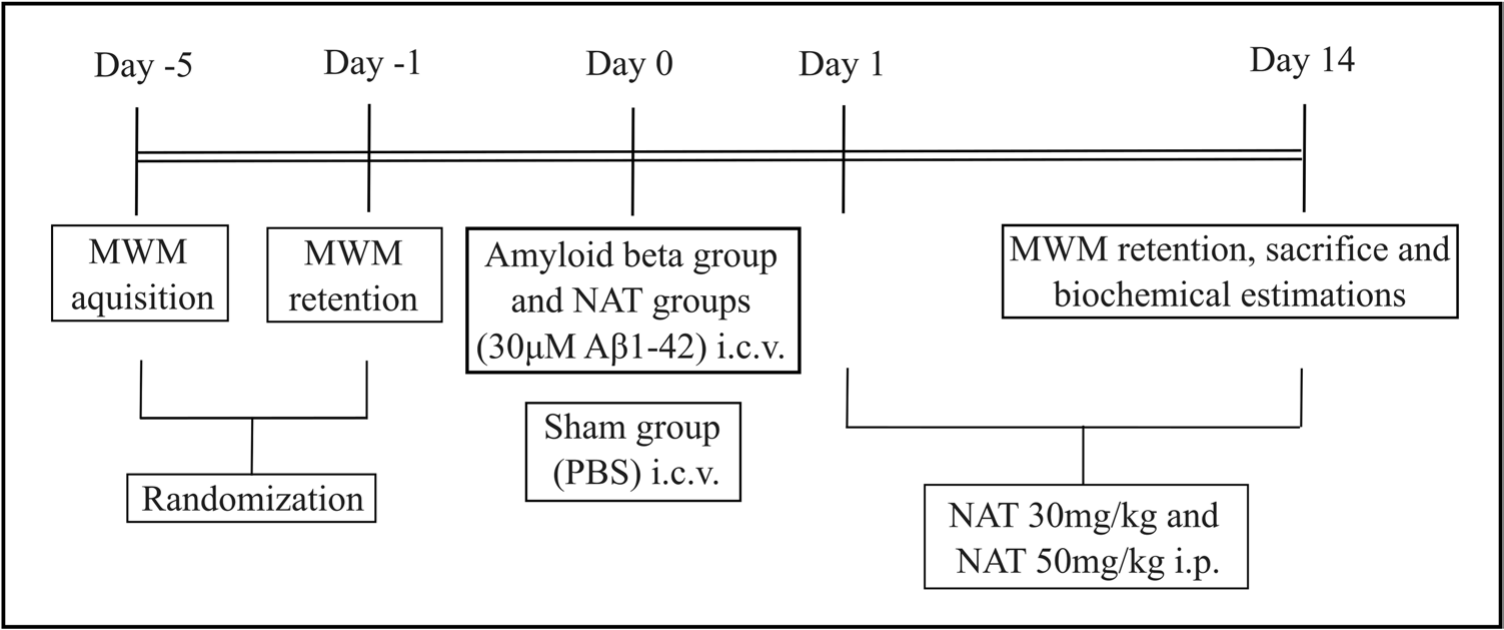
Study design

Before the start of the study, the animals were randomized using MWM, as shown in Fig. 1. The animals with escape latency of less than 20 s and similar average swim speeds were selected for further study to rule out cognitive alterations by birth and locomotor defects, if any. The animals (*n* = 7) were grouped into sham group (PBS), amyloid beta (30 μM Aβ 1-42), amyloid beta + NAT 30 mg/kg, and amyloid beta + NAT 50 mg/kg.

### Administration of Aβ 1-42 Oligomers

The animals were anesthetized using ketamine (80 mg/kg) and xylazine (10 mg/kg). All the animals received respective solutions using a Hamilton Neuros syringe (Hamilton Company, USA); bilateral injections (5 μl/ventricle) were injected 0.8 mm posterior to bregma, 1.5 mm lateral to the sagittal suture, and 3.6 mm below the brain surface [47–49], at the rate of 6 μl/min using Quintessential Stereotaxic Injector with Digital reader (Stoelting Co.). The syringe was guided by StereoDrive (Neurostar, Germany). Two minutes after the injection, the needle was withdrawn. The sham control received PBS, while the disease and treatment groups received 30 μM Aβ 1-42 oligomers. The dose of Aβ 1-42 oligomers was selected based on previous standardization in our lab. The holes were covered using dental cement, and the skin was sutured. A povidone-iodine (10%) solution was applied to the incised skin during the recovery period of 7 days.

### Administration of NAT

Both the doses, NAT 30 mg/kg and NAT 50 mg/kg, were prepared using 0.25% carboxymethylcellulose (CMC) as the suspending agent. The sham control received only 0.25% CMC, while the treatment groups received NAT 30 mg/kg and NAT 50 mg/kg intraperitoneally, daily, for 14 days.

### Morris Water Maze (MWM)

The MWM apparatus comprised a circular pool 150 cm in diameter, which was virtually partitioned into four equal quadrants (A, B, C, D), and a hidden platform of diameter 14 cm was placed in any of the quadrants (D in this case), about 1–2 cm below the water surface. Near the platform, a cue was placed along the inner wall of the pool, and the animals were trained to locate the platform with the aid of that cue. The animals were trained thrice daily with different starting positions in each trial for 4 consecutive days. The inter-trial interval was 30 s. On day 5, the platform was removed, and a retention trial was taken to evaluate the spatial memory [26, 28, 29, 50]. The data was recorded using Any-maze software (Stoelting Co.) through a PC connected to a video camera. After the induction of AD and treatment with NAT, as depicted in Fig. 1, a retention trial on day 14 was taken. The escape latency, path efficiency, number of platform crossings, and swim speed were noted.

### Tissue Preparation for Acetylcholinesterase Activity, Enzyme-Linked Immunosorbent Assay, and Western Blotting

The animals were euthanized, and the hippocampus and frontal cortex were isolated on an ice pack. Immediately, the tissues were homogenized. For ELISA and AChE activity, the tissues were homogenized with cold 1× PBS pH 7.4 in ice-cold conditions and centrifuged in a cooling centrifuge at 10,000 RPM for 12 min to collect a clear supernatant used for the analysis. For western blotting, the tissues were homogenized using RIPA buffer containing protease and phosphatase inhibitor cocktail at 10,000 RPM for 10 min and centrifuged in a cooling centrifuge at 16,000 RPM for 20 min to obtain a clear supernatant. The estimation of total protein was done using a BCA protein assay kit.

### Acetylcholinesterase Activity

The acetylcholinesterase activity in the hippocampus and frontal cortex was determined using Ellman’s technique. A unit of activity (U) corresponds to the enzyme level capable of hydrolyzing 1 μmol of a substrate in a minute [51]. In a 96-well plate, phosphate buffer was added, followed by samples and 5,5′-dithiobis-2-nitrobenzoic acid (DTNB). Then, acetylthiocholine iodide was added, and the resultant yellow color was immediately measured spectrophotometrically at 412 nm for 5 min at an interval of 1 min using the kinetic method. The results are expressed as μM of acetylcholine iodide hydrolyzed/min/mg of protein (U/mg of protein).

### Estimation of Cytokines

The rat TNF-α enzyme-linked immunosorbent assay (ELISA) kit (BMS622) and rat IL-6 ELISA kit (BMS625) obtained from Thermo Fisher Scientific (MA, USA) were used to quantify the cytokine levels in the hippocampus and frontal cortex. The analysis was done as per the manufacturer’s instructions. The standard curve was generated, and the unknown concentrations were extrapolated.

### Estimation of Substance P

Substance P levels of the hippocampus and frontal cortex were measured using an SP ELISA kit (KGE007) from R&D Systems, Inc. (MN, USA). The standard curve was generated, and the unknown concentrations were extrapolated.

### Western Blotting

A protein of 30 μg was run using 12% SDS–PAGE (sodium dodecyl sulfate–polyacrylamide gel electrophoresis) gel. The proteins were then transferred onto a methanol-activated PVDF membrane. A solution of 5% BSA was used to block the membranes for 2 h at room temperature (RT), followed by incubation with primary antibodies like rabbit anti-P-NFκB p65 (1:1000), rabbit anti-NFκB p65 (1:1000), rabbit anti-P-CREB1 (1:1000), rabbit anti-T-CREB1 (1:1000), rabbit anti-P-Tau (1:1000), and rabbit anti-T-Tau (1:1000) at 4 °C overnight. The rabbit anti-alpha-tubulin (1:1000) was used as a control. The membranes were washed using tris-buffered saline (TBST) and incubated with anti-rabbit IgG secondary antibody (1:10,000) for 2 h at RT. The protein bands were visualized using an ECL reagent in a G:BOX gel documentation system (Syngene, UK). Densitometric analysis of the bands was performed using ImageJ software (National Institutes of Health, USA).

### Quantification of NAT in Brain Homogenate

A prominence HPLC system (Shimadzu Corporation, Kyoto, Japan) containing an LC-20AD pump, SIL-20AC HT auto-injector, and SPD-M20A photodiode array detector was used for the determination of NAT in brain homogenate samples. Chromatographic separation of NAT was performed using the kinetex C_18_ column (250 × 4.6 mm, 5 μm). We used isocratic elution mode for brain homogenate samples with the mobile phase ratio of 43:57% v/v of methanol and 25 mM phosphate buffer (pH 3.5) with 0.1% TEA. Both the mobile phases were passed through a 0.22-μm PTFE filter (Whatman, Inc., UK) and sonicated for 20 min in a bath sonicator (Microclean-101, Oscar ultrasonic, India) before use. The flow rate was adjusted to 1 ml/min with 40-μl injection volume at a wavelength of 280 nm. The protein precipitation extraction technique was employed to extract NAT in brain samples using an identical amount of chilled acetonitrile. Raloxifene HCl served as an internal standard. The bioanalytical method for brain homogenate was validated following the USFDA protocol.

### Simulation of Plasma NAT Levels Using GastroPlus™

To predict the plasma levels of NAT in rats, we employed GastroPlus™ simulation software (version 9.7, Simulation Plus Inc., USA). The structure of NAT was imported, and with the aid of the ADMET predictor module, the software generated the physicochemical properties of NAT. These parameters were used to predict the plasma concentration of NAT suspension via oral and i.v routes. These values were extrapolated to levels of NAT obtained in the *in vivo* rat brain samples.

### Statistical Analysis

GraphPad Prism 8.0.2 was employed for statistical analysis (Graph Pad Software Inc., San Diego, CA, USA). The results were presented as mean ± standard error of the mean (SEM). One-way analysis of variance (ANOVA) was used to compare the groups, followed by Tukey’s multiple comparison test. Results were significant if p<0.05, p<0.01, p<0.001 and p<0.0001.

## Results

### Visualization of the Aβ 1-42 Oligomers Using Atomic Force Microscopy

**Fig. 2:**
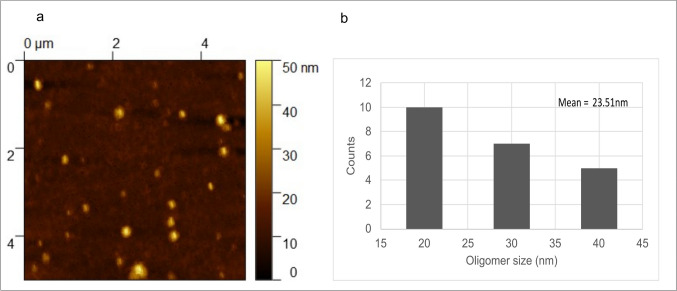
AFM imaging of Aβ 1-42 oligomers. The Aβ 1-42 oligomers were placed on a clean glass coverslip and were subjected to AFM analysis. **a** The AFM image of Aβ 1-42 oligomers. The image was obtained in a scan range of 5 μm × 5 μm with a *Z* range of 50 nm. **b** The size distribution and mean size of oligomers

The incubation of Aβ 1-42 peptide with 1× PBS for 72 h at 4 °C facilitated the formation of Aβ 1-42 oligomers. They appeared as globular-shaped particles, as seen in Fig. 2a. The Aβ 1-42 oligomer size distribution analyses revealed that these oligomers ranged from 14 to 40 nm in size with an average size of 23.51 nm, as shown in Fig. 2b.

### The Protective Action of NAT on Spatial Memory in Aβ 1-42-Induced Cognitive Decline in the MWM

**Fig. 3:**
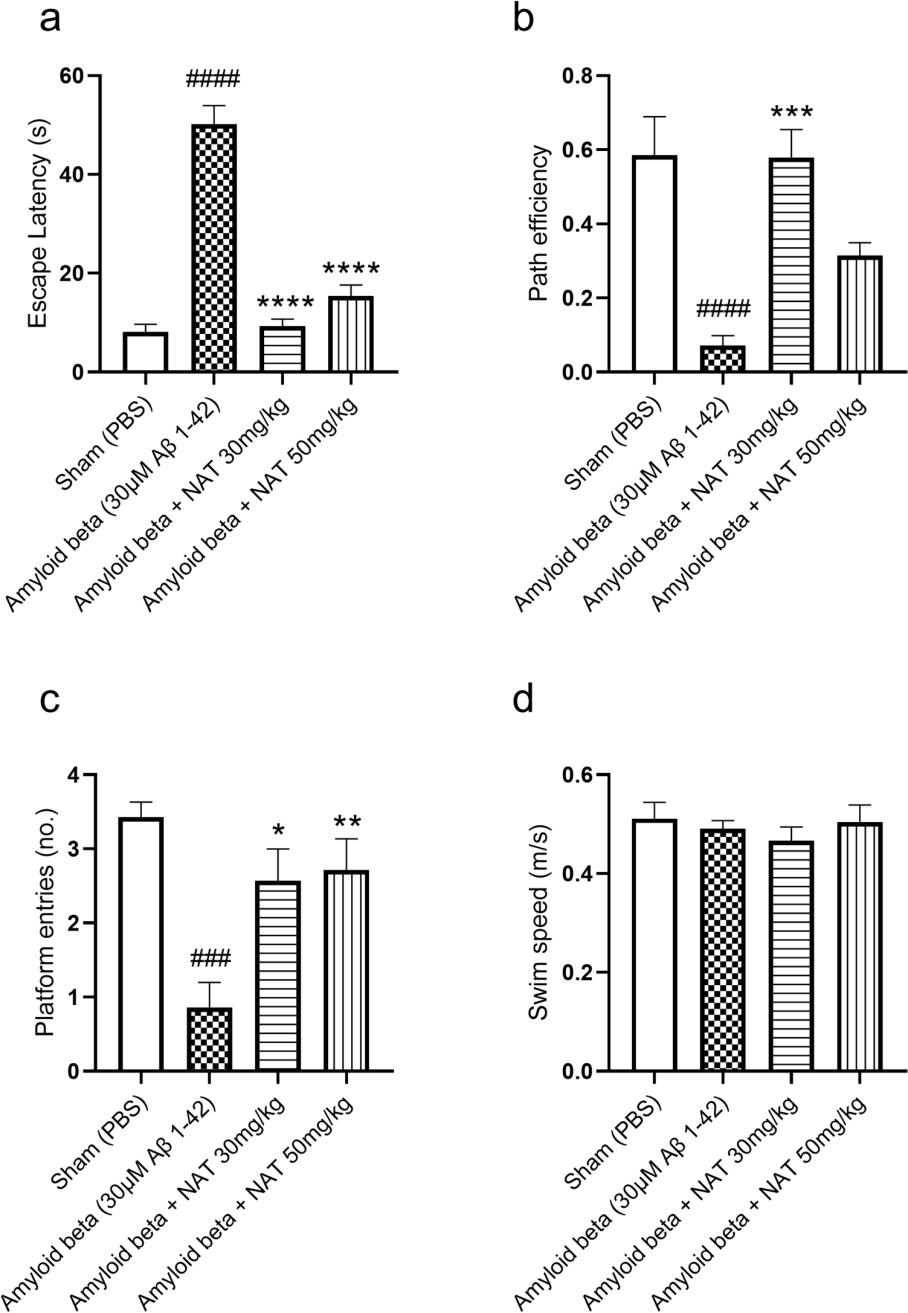
Effect of NAT on the spatial memory in rats with Aβ 1-42-induced cognitive decline. The animals were subjected to a probe trial on the fifth day in MWM. Parameters like escape latency (**a**), path efficiency (**b**), platform entries (**c**), and swim speed (**d**) were assessed. Data was analyzed using one-way ANOVA followed by Tukey’s multiple comparison test. ###*p* <0.001, ####*p* < 0.0001 vs. sham group; *p < 0.05, **p < 0.01, ***p < 0.001, ****p < 0.0001 vs. amyloid beta group

We investigated the effect of NAT 30 mg/kg and NAT 50 mg/kg in Aβ 1-42-treated rats in MWM, as shown in Fig. 3a–d. We observed that the Aβ 1-42 treatment in the amyloid beta group caused a significant elevation in escape latency (50.16 ± 3.766 vs. 8.143 ± 1.518, *p* < 0.0001), a significant reduction in path efficiency (0.07157 ± 0.02655 vs. 0.5856 ± 0.1032, *p* < 0.0001), and platform entries (0.8571 ± 0.3401 vs. 3.429 ± 0.2020, *p* < 0.001) as compared to the sham-treated animals. We then compared the results of the treatment groups with the amyloid beta group to understand the effect of the NAT 30 mg/kg and 50 mg/kg in diseased conditions. The escape latency in amyloid beta + NAT 30 mg/kg (9.257 ± 1.459 vs. 50.16 ± 3.766, *p* < 0.0001) and amyloid beta + NAT 50 mg/kg (15.44 ± 2.179 vs. 50.16 ± 3.766, *p* < 0.0001, *F* (3, 24) = 67.56) showed a profound reduction as compared to the amyloid beta group. Similarly, a marked increase in the platform entries was observed in amyloid beta + NAT 30 mg/kg (2.571 ± 0.4286 vs. 0.8571 ± 0.3401, *p* < 0.05) and amyloid beta + NAT 50 mg/kg (2.714 ± 0.4206 vs. 0.8571 ± 0.3401, *p* < 0.01, *F* (3, 24) = 9.197) in comparison to amyloid beta group. Interestingly, only amyloid beta + NAT 30 mg/kg showed a significant rise in path efficiency (0.5787 ± 0.07567 vs. 0.07157 ± 0.02655, *p* < 0.001, *F* (3, 24) = 13.19) in comparison to the amyloid beta group. There were no significant changes in the swim speed in the NAT treatment groups, suggesting that none of the interventions affected the locomotion of the animals.

### Treatment with NAT Mediates the Downregulation of TNF-α, IL-6, and SP in the Hippocampus and Frontal Cortex of Aβ 1-42-Treated Animals

**Fig. 4:**
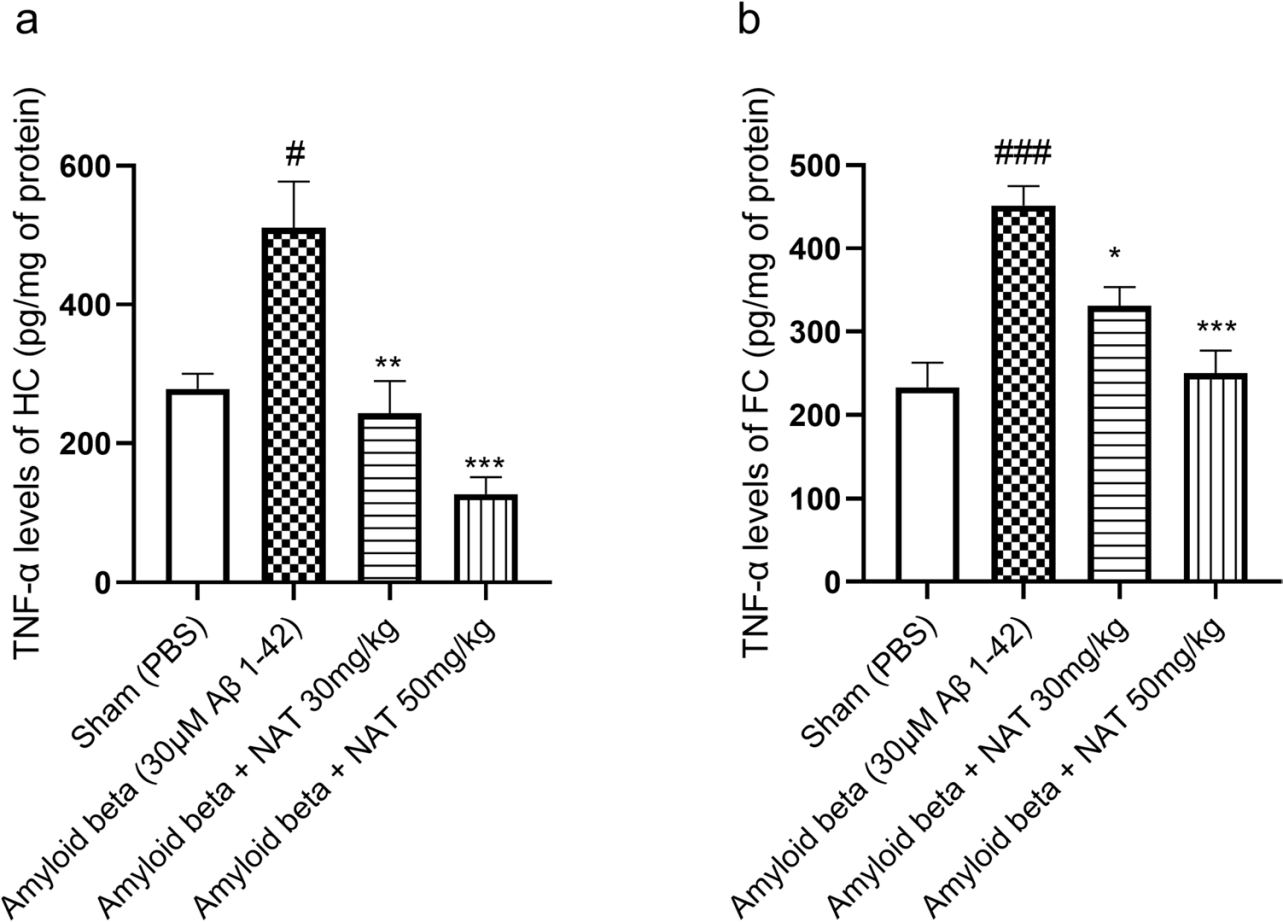
Effect of NAT treatment on the levels of TNF-α. The hippocampal and frontal cortex samples were analyzed for expression of TNF-α. The representative figures depict **a** TNF-α levels in the hippocampus and **b** TNF-α levels in the frontal cortex. Data was analyzed using one-way ANOVA followed by Tukey’s multiple comparison test. #*p* < 0.05, ###*p* < 0.001 vs. sham group; *p  < 0.05, **p < 0.01, ***p < 0.001 vs. amyloid beta group. Legend—TNF-α, tumor necrosis factor; HC, hippocampus; FC, frontal cortex

The TNF-α is known to hamper the retrieving and consolidation of the spatial and contextual fear memories regulated by the hippocampus [52]. Our results revealed that the hippocampal region of the amyloid beta group (510.7 ± 66.55 vs. 278.2 ± 21.91, *p* < 0.05) expressed higher levels of TNF-α than the sham group. Interestingly, the amyloid beta + NAT 30 mg/kg (243.1 ± 46.52 vs. 510.7 ± 66.55, *p* < 0.01) and amyloid beta + NAT 50 mg/kg (127.2 ± 24.56 vs. 510.7 ± 66.55, *p* < 0.001, *F* (3, 12) = 13.47) significantly reduced the TNF-α levels in comparison to the amyloid beta group as shown in Fig. 4a.

Similarly, in the frontal cortex, there was a profound increase in TNF-α in the amyloid beta group (451.6 ± 23.35 vs. 233.1 ± 29.38, *p* < 0.001) compared to the sham group. The treatment groups, amyloid beta + NAT 30 mg/kg (330.9 ± 22.81 vs. 451.6 ± 23.35, *p* < 0.05) and amyloid beta + NAT 50 mg/kg (250.4 ± 26.87 vs. 451.6 ± 23.35, *p* < 0.001, *F* (3, 12) = 14.98), showed a marked reduction in the TNF-α levels as shown in Fig. 4b. This could indicate that, upon exposure to Aβ 1-42, there could be an elevation in the TNF-α levels in the hippocampus and frontal cortex regions due to an increase in neuroinflammation. This was seen to be reversed by the NAT treatments.

**Fig. 5:**
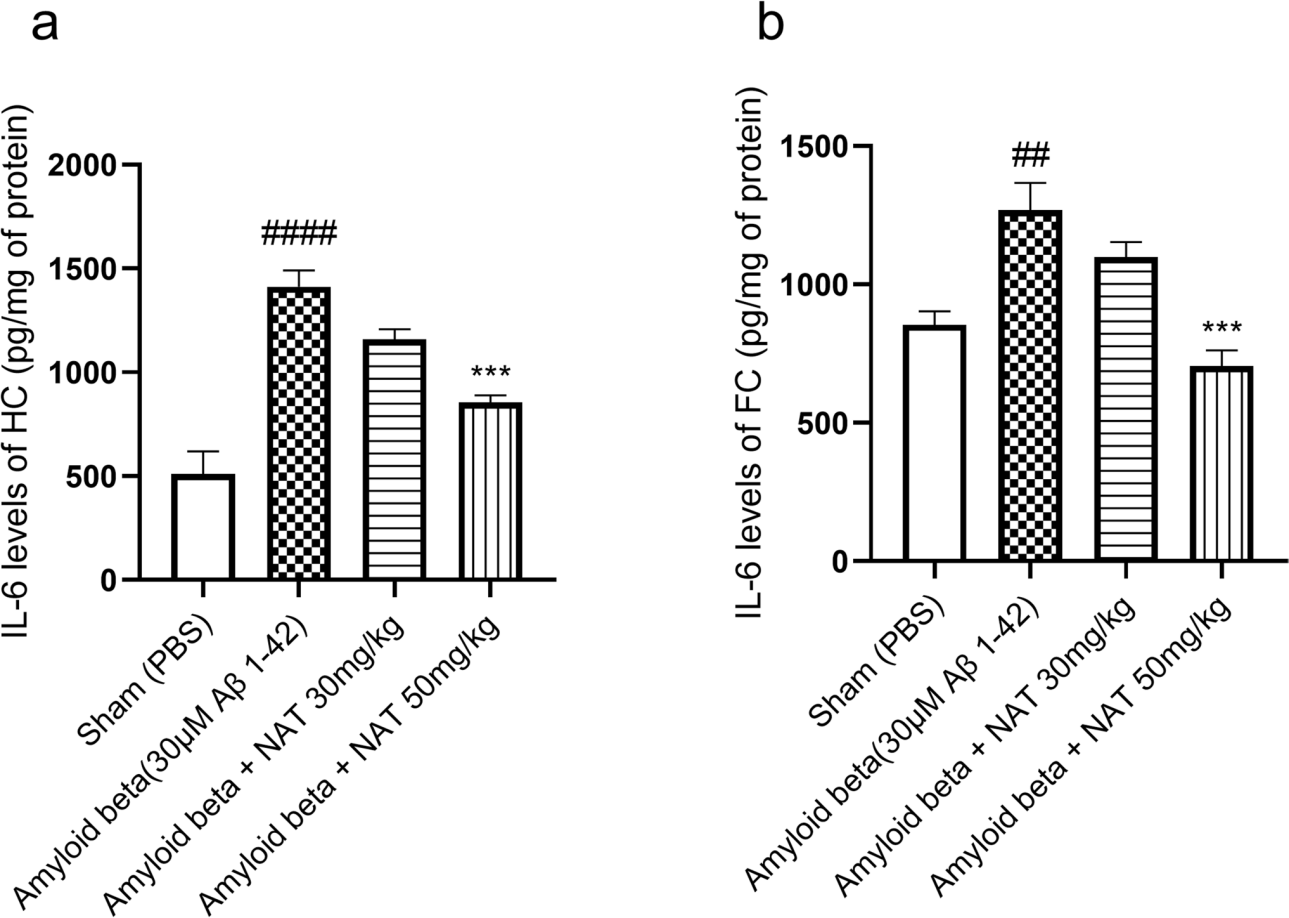
Effect of NAT treatment on the levels of IL-6. The hippocampal and frontal cortex samples were analyzed for expression of IL-6. Representative figures depict **a** the levels of IL-6 in the hippocampus and **b** the levels of IL-6 in the frontal cortex. Data was analyzed using one-way ANOVA followed by Tukey’s multiple comparison test. ####*p* < 0.0001, ##*p* < 0.01 vs. sham group; ***p < 0.001 vs. amyloid beta group. Legend—IL-6, interleukin-6; HC, hippocampus; FC, frontal cortex

Another vital role player in neuroinflammation is the IL-6 [53]. The activation of the IL-6 pathway in the CNS has been linked with cognitive imbalance in AD [54]. In this context, we analyzed the levels of IL-6. It was seen that, in the hippocampus, the levels of IL-6 in the amyloid beta group (1412 ± 78.45 vs. 511.1 ± 107.9, *p* < 0.0001) showed a significant elevation in comparison to the sham group. Surprisingly, the amyloid beta + NAT 30 mg/kg did not significantly affect IL-6 levels. Nevertheless, the amyloid beta + NAT 50 mg/kg group (855.4 ± 33.06 vs. 1412 ± 78.45, *p* < 0.001, *F* (3, 12) = 28.67) showed a significant reduction in IL-6 levels in comparison to the amyloid beta group, as shown in Fig. 5a.

Similarly, in the frontal cortex, a profound elevation in IL-6 levels was observed in the amyloid beta group (1268 ± 99.03 vs. 854.5 ± 48.81, *p* < 0.01) compared to the sham group. Interestingly, a marked reduction in IL-6 levels was noted in the amyloid beta + NAT 50 mg/kg group (705.3 ± 56.22 vs. 1268 ± 99.03, *p* < 0.001, *F* (3, 12) = 13.75) in comparison to the amyloid beta group as shown in Fig. 5b.

**Fig. 6:**
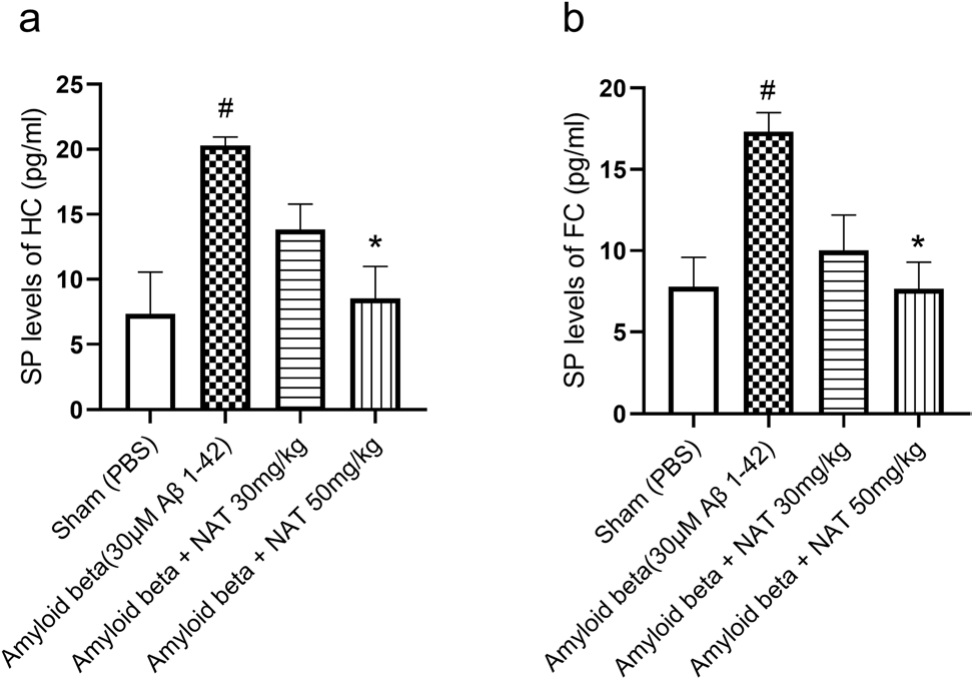
Effect of NAT treatment on the levels of SP. The hippocampal and frontal cortex samples were analyzed for expression of SP. The representative figures depict **a** SP levels in the hippocampus and **b** SP levels in the frontal cortex. Data was analyzed using one-way ANOVA followed by Tukey’s multiple comparison test. #*p* < 0.05 vs. sham group; *p < 0.05 vs. amyloid beta group. Legend—SP, substance P; HC, hippocampus; FC, frontal cortex

Our results demonstrated that the hippocampal region of the amyloid beta group (20.30 ± 0.6438 vs. 7.346 ± 3.200, *p* < 0.05) had significantly elevated SP levels compared to the sham group. On the contrary, the amyloid beta + NAT 50 mg/kg (8.536 ± 2.460 vs. 20.30 ± 0.6438, *p* < 0.05, *F* (3, 8) = 6.826) was markedly able to reduce these levels in comparison to the amyloid beta group as shown in Fig. 6a.

Similarly, in the frontal cortex, the hippocampus of the amyloid beta group (17.33 ± 1.170 vs. 7.783 ± 1.804, *p* < 0.05) demonstrated higher SP levels than the sham. Even though there were no significant changes in the amyloid beta + NAT 30 mg/kg group, interestingly, the amyloid beta + NAT 50 mg/kg group (7.651 ± 1.634 vs. 17.33 ± 1.170, *p* < 0.05, *F* (3, 8) = 6.922) showed a profound reduction in SP levels as shown in Fig. 6b.

These findings suggest the involvement of SP in the diseased states, possibly via the NK1R axis. These findings also suggest that treatment with NAT could exert protective actions in neurodegenerative diseases like AD.

### Administration of NAT Regulates the Expression of NFκB p65, CREB1, and Tau Proteins

**Fig. 7:**
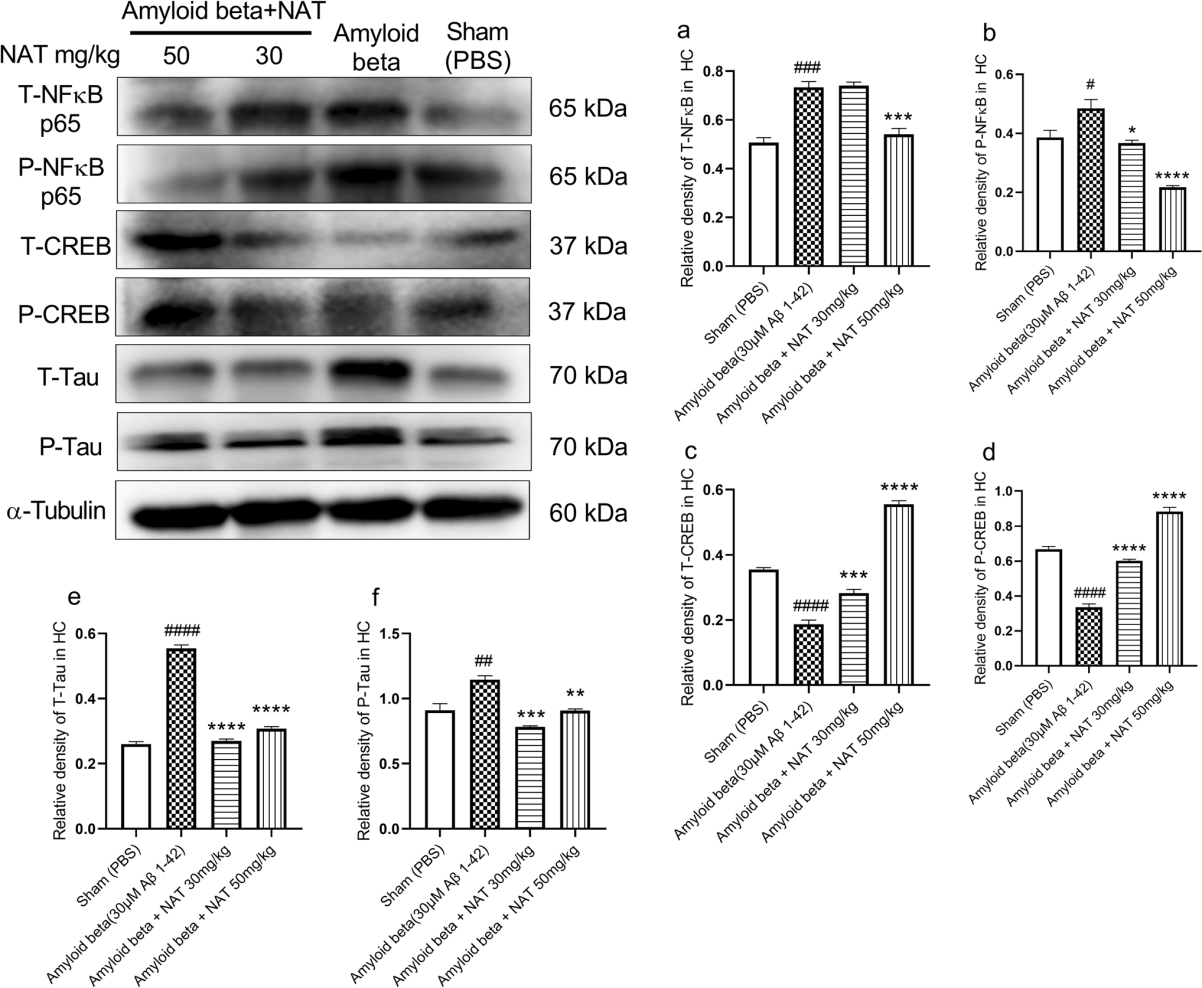
Effect of NAT on NFκB p65, CREB1, and Tau expression levels in the hippocampus of Aβ 1-42-treated rats. Western blotting technique was used to evaluate the relative expression of markers like NFκB p65, CREB1, and Tau in the hippocampus. The phosphorylated and unphosphorylated levels of NFκB p65, CREB1, and Tau were normalized to the corresponding α-tubulin values. (a–f) The relative expression of these proteins in the hippocampus. Data was analyzed using one-way ANOVA followed by Tukey’s multiple comparison test. ####*p*<0.0001, ###*p*<0.001, ##*p*<0.01, #*p*<0.05 vs. sham group; *p<0.05, **p<0.01, ***p<0.001, and ****p<0.0001 vs. amyloid beta group. Legend—T-NFκB, total nuclear factor kappa B; P-NFκB, phosphorylated nuclear factor kappa B; T-CREB1, total cyclic AMP response element-binding protein 1; P-CREB1, phosphorylated cyclic AMP response element-binding protein 1; T-Tau, total Tau; P-Tau, phosphorylated Tau; α-tubulin, alpha tubulin; HC, hippocampus

To investigate the effect of NAT treatments on neuroinflammation induced by Aβ 1-42 oligomers, we analyzed T-NFκB p65 and P-NFκB p65 in both hippocampal and frontal cortex samples. All the values were expressed as the relative density of the target proteins, normalized to α-tubulin densities. Efficient bidirectional communication between hippocampal formation and the prefrontal cortex is instrumental in regulating various processes related to cognition and emotions [24]. The NFκB is known to be a central regulator of inflammation in the conditions of AD [55]. It stimulates pro-inflammatory mediators and downregulates neuroprotective components in the CNS [56]. In the hippocampus, the relative expression of T-NFκB p65 (0.7334 ± 0.02352 vs. 0.5073 ± 0.01961, *p* < 0.001) and P-NFκB p65 (0.4846 ± 0.02994 vs. 0.3860 ± 0.02418, *p* < 0.05) in the amyloid beta group was significantly higher in comparison to the sham group as shown in Fig. 7a and b. Interestingly, the amyloid beta + NAT 50 mg/kg group showed a significant reduction in the expression of T-NFκB p65 (0.5410 ± 0.02405 vs. 0.7334 ± 0.02352, *p* < 0.001, *F* (3, 8) = 35.74) as compared to the amyloid beta group. However, the levels of P-NFκB p65 showed a marked reduction in both amyloid beta + NAT 30 mg/kg (0.3670 ± 0.009652 vs. 0.4846 ± 0.02994, *p* < 0.05) and amyloid beta + NAT 50 mg/kg groups (0.2174 ± 0.005163 vs. 0.4846 ± 0.02994, *p*<0.0001, *F* (3, 8) = 30.41).

**Fig. 8:**
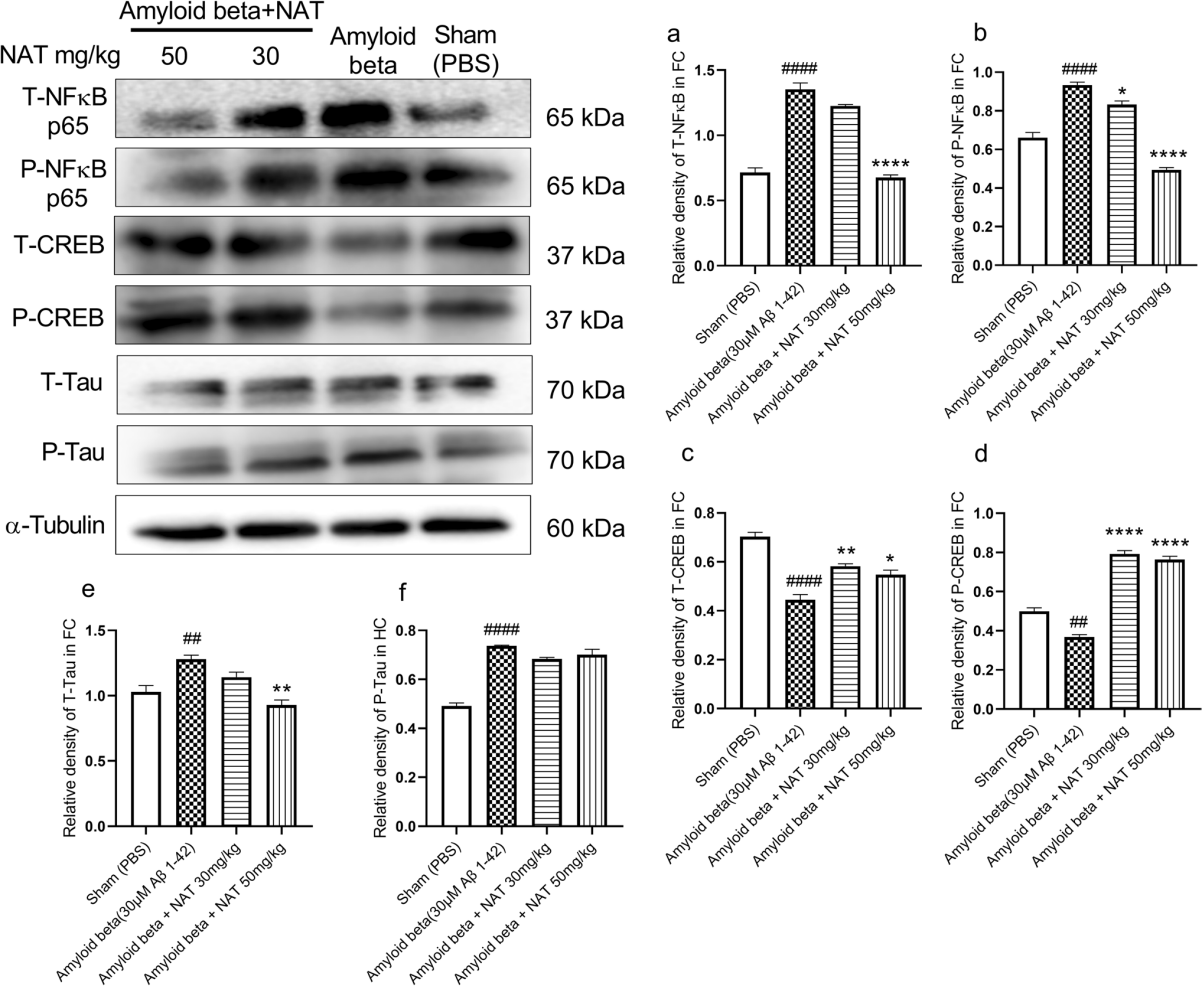
Effect of NAT on NFκB p65, CREB1, and Tau expression levels in the frontal cortex of Aβ 1-42-treated rats. Western blotting technique was used to evaluate the relative expression of markers like NFκB p65, CREB1, and Tau in the frontal cortex. The phosphorylated and unphosphorylated levels of NFκB p65, CREB1, and Tau were normalized to the corresponding α-tubulin values. (a–f) The relative expression of these proteins in the frontal cortex. Data was analyzed using one-way ANOVA followed by Tukey’s multiple comparison test. ####*p*<0.0001, ##*p*<0.001 vs. sham group; *p<0.05, **p<0.01, and ****p<0.0001 vs. amyloid beta group. Legend—T-NFκB, total nuclear factor kappa B; P-NFκB, phosphorylated nuclear factor kappa B; T-CREB1, total cyclic AMP response element-binding protein 1; P-CREB1, phosphorylated cyclic AMP response element-binding protein; T-Tau, total Tau; P-Tau, phosphorylated Tau; α-tubulin, alpha tubulin; FC, frontal cortex

In the frontal cortex, the amyloid beta group showed significantly upregulated expression of T-NFκB p65 (1.353 ± 0.04876 vs. 0.7158 ± 0.03443, *p*<0.05) and P-NFκB p65 (0.9341± 0.01475 vs. 0.6610± 0.02689, *p* < 0.05) in comparison to the sham group as shown in Fig. 8a and b. The amyloid beta + NAT 30 mg/kg group was significantly able to reduce the expression of only P-NFκB p65 (0.8329 ± 0.01842 vs. 0.9341± 0.01475, *p* < 0.05) in comparison to the amyloid beta group. Interestingly, the amyloid beta + NAT 50 mg/kg group showed a marked reduction in both T-NFκB p65 (0.6769 ± 0.02020 vs. 1.353 ± 0.04876, *p* < 0.05, *F* (3, 8) = 117.4) and P-NFκB p65 (0.4948 ± 0.01114 vs. 0.9341± 0.01475, *p* < 0.05, *F* (3, 8) = 106.7) in comparison to the amyloid beta group.

This could suggest the upregulation of NFκB p65 in the diseased conditions and the concurrent role of NAT in their reduction in Aβ pathologies.

The CREB1 is a crucial transcription factor in regulating neuronal growth and its differentiation/ proliferation, accelerating synaptic plasticity, and influencing spatial memory formation and long-term memory [57]. Our evaluation revealed that, in the hippocampus, T-CREB1 (0.1871 ± 0.01285 vs. 0.3551 ± 0.006253, *p*<0.001) and P-CREB1 (0.3352 ± 0.01972 vs. 0.6691 ± 0.01491, *p*<0.001) expression in the amyloid beta group was significantly reduced in comparison to the sham group as shown in Fig. 7c and d. In the amyloid beta + NAT 30 mg/kg, both T-CREB1 (0.2828 ± 0.01128 vs. 0.1871 ± 0.01285, *p*<0.001) and P-CREB1 levels (0.6019 ± 0.009036 vs. 0.3352 ± 0.01972, *p*<0.001) were significantly elevated as compared to the amyloid beta group. The amyloid beta + NAT 50 mg/kg showed similar results in T-CREB1 (0.5555 ± 0.01052 vs. 0.1871 ± 0.01285, *p*<0.001, *F* (3, 8) = 220.8) and P-CREB1 (0.8835 ± 0.02348 vs. 0.01285, *p*<0.001, *F* (3, 8) = 164.2) levels as compared to the expression in the amyloid beta group.

Upon analysis of the CREB1 levels in the frontal cortex of the amyloid beta group, we observed a profound reduction of T-CREB1 (0.4447 ± 0.02166 vs. 0.7029 ± 0.01827, *p*<0.001) and P-CREB1 (0.3678 ± 0.01290 vs. 0.4997 ± 0.01772, *p*<0.01) in comparison to the sham group as shown in Fig. 8c and d. On the contrary, a significant rise in the levels of T-CREB1 (0.5823 ± 0.009780 vs. 0.4447 ± 0.02166, *p*<0.01) and P-CREB1 (0.7928 ± 0.01647 vs. 0.3678 ± 0.01290, *p*<0.0001) levels was observed in the amyloid beta + NAT 30 mg/kg group on comparing with the amyloid beta group. As expected, these results were also consistent in the amyloid beta + NAT 50 mg/kg group, which similarly showed significant elevation of T-CREB1 (0.5479 ± 0.01868 vs. 0.4447 ± 0.02166, *p*<0.05, *F* (3, 8) = 36.36) and P-CREB1 (0.7640 ± 0.01618 vs. 0.3678 ± 0.01290, *p*<0.0001, *F* (3, 8) = 168.3) levels against the levels in the amyloid beta group.

The Tau protein is a microtubule-associated protein that provides structural stability to microtubules. However, in AD conditions, it undergoes abnormal hyperphosphorylation, leading to the disruption of microtubules and the formation of NFTs, further worsened by Aβ [58]. It was seen that, in the hippocampus, the relative expression of T-Tau levels (0.5542 ± 0.01046 vs. 0.2601± 0.007827, *p*<0.0001) and P-Tau levels (1.145 ± 0.03038 vs. 0.9109 ± 0.05021, *p* < 0.001) expressed in the amyloid beta group was markedly increased as compared to the sham group as seen in Fig. 7e and f. It was pretty interesting to discover that the amyloid beta + NAT 30 mg/kg group significantly reduced the levels of T-Tau (0.2698 ± 0.006129, *p* < 0.0001 vs. 0.5542 ± 0.01046) and P-Tau (0.7813 ± 0.009415 vs. 1.145 ± 0.03038, *p*<0.001) in comparison to the amyloid beta group. Similarly, the amyloid beta + NAT 50 mg/kg group demonstrated a reduction in the T-Tau levels (0.3079 ± 0.005680 vs. 0.5542 ± 0.01046, *p*<0.0001, *F* (3, 8) = 321.4) and P-Tau levels (0.9085 ± 0.01240 vs. 1.145 ± 0.03038, *p*<0.01, *F* (3, 8) = 25.02) in comparison to amyloid beta groups.

The relative densities of T-Tau (1.280 ± 0.03107 vs. 1.030 ± 0.04874, *p* <0.01) and P-Tau 0.7367 ±0.002717 vs. 0.4913 ± 0.01274, *p*<0.0001, *F* (3, 8) = 70.07) in the frontal cortex of the amyloid beta group were significantly higher in comparison to the sham group as shown in Fig. 8e and f. Surprisingly, the amyloid beta + NAT 30 mg/kg showed no significant alterations. In contrast, the amyloid beta + NAT 50 mg/kg group was able to significantly reduce the expression of only T-Tau (0.9300 ± 0.03710 vs. 1.280 ± 0.03107, *p*<0.01, *F* (3, 8) = 14.40) as compared to the amyloid beta group.

These results could suggest that the AD conditions induced by the administration of Aβ 1-42 oligomers might have triggered the alterations in these proteins. Thus, the administration of NAT could have exerted protective effects, suggesting the neuroprotective role of NAT in AD conditions.

### NAT Treatment Mediates the Downregulation of AChE Activity in the Hippocampus and Frontal Cortex

**Fig. 9:**
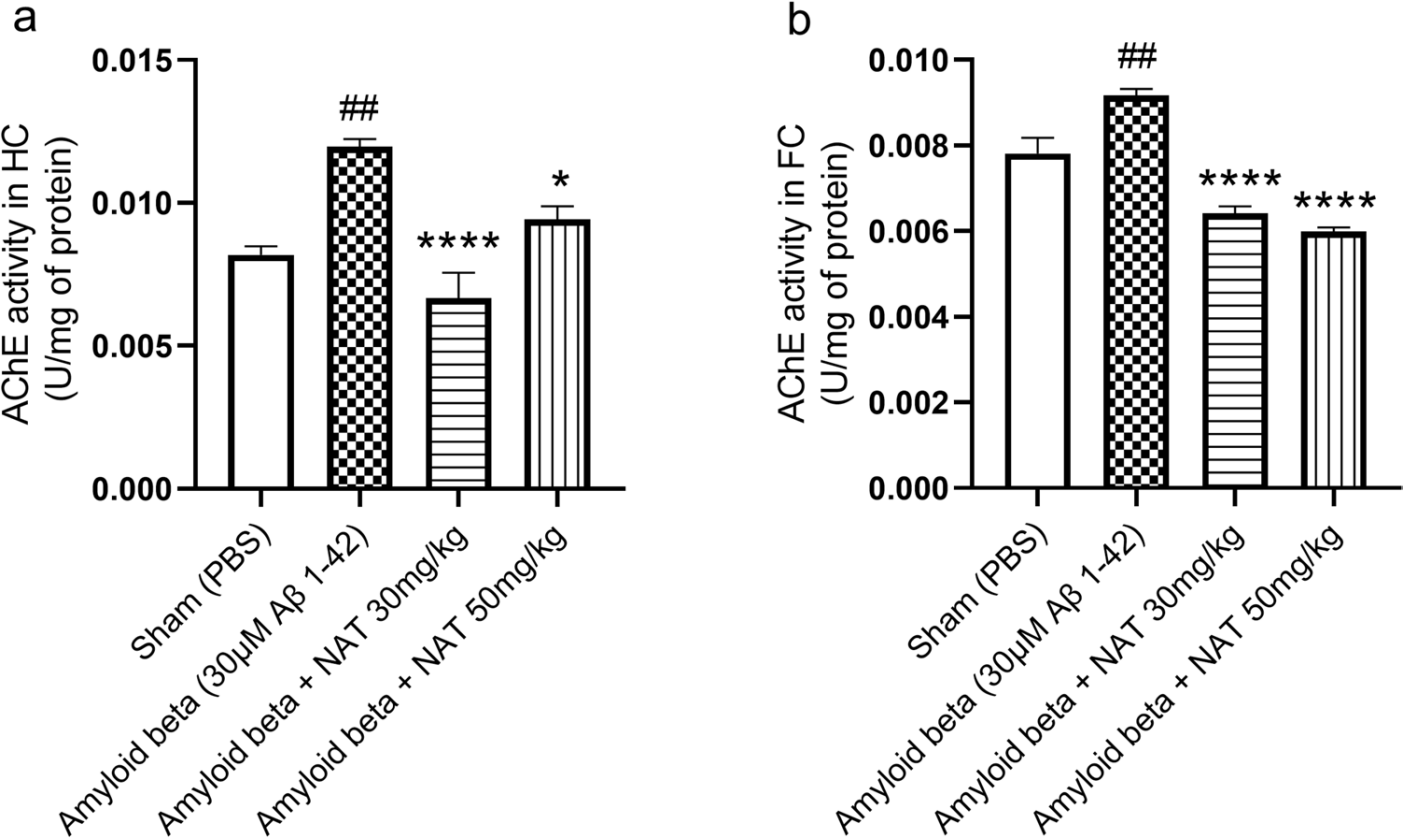
Effect of NAT treatment on the levels of AChE activity. The activity of AChE in the hippocampus and frontal cortex was evaluated to understand its alterations in the presence of Aβ 1-42. The representative figure shows AChE activity **a** in the hippocampus and **b** in the frontal cortex. Data was analyzed using one-way ANOVA followed by Tukey’s multiple comparison test. ##*p*<0.01 vs. sham group; *p<0.05 and ****p<0.0001 vs. amyloid beta group. Legend—AChE, acetylcholinesterase; HC, hippocampus; FC, frontal cortex

Acetylcholinesterase hydrolyzes Ach into choline and acetic acid and terminates synaptic transmission through acetylcholine. AChE inhibition could thus increase the levels of acetylcholine and provide protective effects against cognitive decline seen in conditions like AD [59]. AChE activity is lower in most AD brain regions but increases within and around amyloid plaques [60]. Our analysis was in line with the idea that the action of AChE was profoundly higher in the hippocampal region of the amyloid beta group (0.01196 ± 0.0002748 vs. 0.008180 ± 0.0002981, *p*< 0.01) in comparison to the levels estimated in the sham group. We further wanted to assess the effect of NAT treatment on AChE activity. As expected, the amyloid beta + NAT 30 mg/kg (0.006670 ± 0.0008817 vs. 0.01196± 0.0002748, *p*< 0.0001) and amyloid beta + NAT 50 mg/kg group (0.009420 ± 0.0004594 vs. 0.01196± 0.0002748, *p*< 0.05, *F* (3, 12) = 17.40) showed a remarkable reduction in the levels of AChE in comparison to the amyloid beta group, as shown in Fig. 9a.

AChE activity extends to the hippocampus and the frontal cortex in neurodegenerative conditions like AD. We observed that, in the frontal cortex, AChE levels increased significantly in the amyloid beta group (0.009173 ± 0.0001497 vs. 0.007810 ± 0.0003695, *p*< 0.01) when correlated with the sham group levels, as shown in Fig. 9b. It was not surprising to see that the amyloid beta + NAT 30 mg/kg (0.006410 ± 0.0001721 vs. 0.009173 ± 0.0001497, *p*<0.0001) and amyloid beta + NAT 50 mg/kg group (0.005985 ± 0.0001044 vs. 0.009173 ± 0.0001497, *p*<0.0001, *F* (3, 12) = 41.97) significantly reduced the levels of AChE as compared to the amyloid beta group, suggesting their neuroprotective role in the conditions of AD.

### Quantification of NAT in the In Vivo Rat Brain

**Fig. 10:**
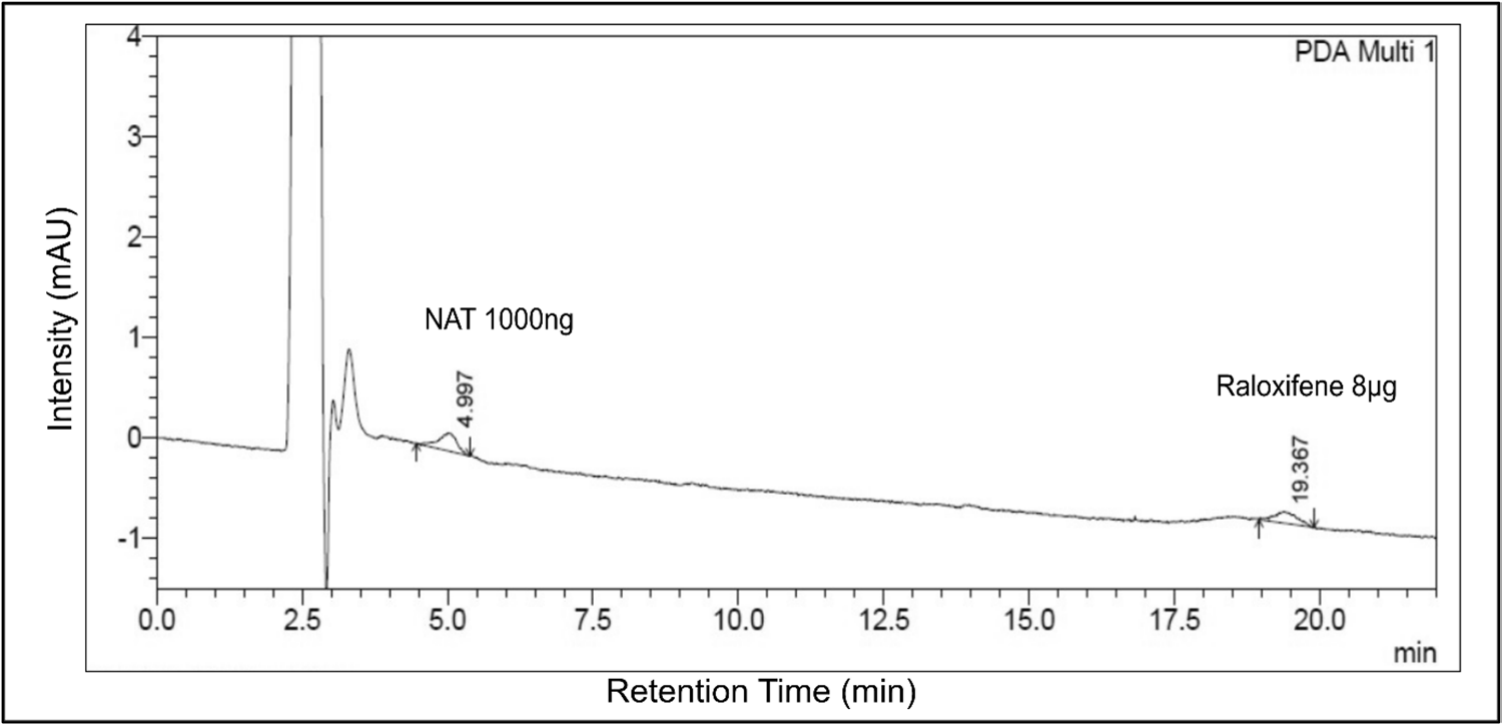
HPLC chromatogram for lowest limit of quantification of brain homogenate method. This figure describes the chromatogram obtained for the lowest limit of quantification for the method developed to evaluate NAT in the brain at a wavelength of 280 nm

The HPLC method for detecting NAT in brain homogenate samples showed a retention time of 4.9 min, having linearity from 1000 to 3200 ng/ml (*R*^2^ = 0.9994). Raloxifene served as an internal standard, showing a retention of 19.367 min. The LLOQ chromatogram for the brain homogenate method is shown in Fig. 10.

Upon analysis of the concentrations of NAT in the brain, it is interesting that no peak was observed at the retention time of NAT, which implies the levels of NAT could be lesser than the lowest limit of quantification of our validated method.

### GastroPlus Mediated Simulation of NAT levels in Rat Plasma

**Fig. 11:**
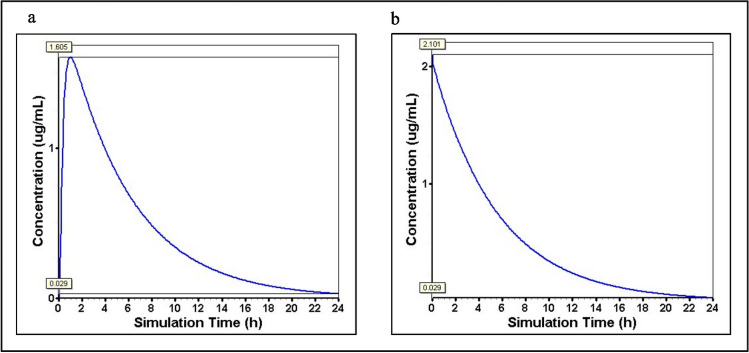
Plasma concentration of NAT as predicted by GastroPlus software. The GastroPlus software predicts the concentration of NAT in the plasma. **a** The predicted plasma concentrations of NAT upon administration of NAT 50 mg/kg dose to rat via oral route; **b** the plasma concentrations of NAT upon administration of NAT 50 mg/kg dose to rat via intravenous route

The NAT concentration in rat brains was undetected in our *in vivo* analysis, which may be attributed to the less sensitivity of the developed HPLC method having the lowest quantification (LLOQ) limit of 1000 ng, as mentioned before. Therefore, using GastroPlus, we simulated the NAT levels in the rat plasma to correlate with values to the brain levels of NAT. The GastroPlus software predicted the plasma Cmax of NAT for oral suspension as 1.605 μg/ml and 2.101 μg/ml for i.v bolus, for a dose of 50 mg/kg administered orally/IV bolus, respectively, as shown in Fig. 11a and b. These values corroborate that NAT concentrations in the brain may be lower than 1 μg/ml.

## Discussion

The complexity of AD brings a whole lot of challenges in the development of effective therapeutics. The therapies offering symptomatic relief have been approved but have not successfully managed AD [61]. This can be attributed to the pathological complexity of AD. Therefore, treatments that delay disease progression and offer a wide range of benefits in the multi-pathological conditions of AD are essential. Our study primarily focuses on evaluating the neuroprotective effect of a peptide, NAT, in ameliorating the neuroinflammation-induced cognitive decline in AD-like conditions. We attempted to develop AD-like conditions in Wistar rats by i.c.v. administration of Aβ 1-42 oligomers. We noticed that oligomeric forms of Aβ 1-42-induced neurotoxicity, causing hampered cognitive function, marked elevation in neuroinflammatory markers and upregulation of Tau, SP, and AChE signaling accompanied by a decline in CREB1 signaling. NAT treatments offered neuroprotection and improved cognition in rats, suggesting its neuroprotective role in AD-like conditions. We also attempted to estimate the concentration of NAT in the CNS, responsible for exerting such beneficial effects. However, NAT remained undetected in brain homogenates, possibly due to its levels being lesser than HPLC method detection limits.

The Aβ oligomers are essential to exert toxicity in the CNS [62]. Nevertheless, its morphological dimensions are crucial in exerting this toxicity [63]. In the tapping mode, the tip used to scan the sample oscillates vertically, lowering the friction load exerted on the specimen and providing the morphological characteristic with lesser distortion [64]. This could be why the tapping mode is employed, as seen in previous Aβ characterization studies [64–67]. Similarly, our study employed tapping mode to visualize the Aβ 1-42 oligomers. It is understood that Aβ 1-42 oligomers appear spherical, and the 8–15 nm size range is essential to exert toxicity [13, 14]. Similarly, our Aβ 1-42 oligomers also belonged to a similar size range.

In AD, as cognitive decline involves impairment in spatial memory, we intended to evaluate the same using MWM. Various parameters like escape latency, path efficiency, and number of platform entries were used to evaluate cognitive decline in MWM [68–70]. As previously reported, one way of the many ways in which Aβ 1-42 could hamper the hippocampal region would be by disruption of neuronal networks in the region of CA1 and CA3, along with the dentate gyrus [71]. This is combined with the conditions of concurrent neuroinflammation. Therefore, the parameters assessed in MWM could reflect these conditions’ consequences. The escape latency measures the time the animal takes to navigate to the platform [70]. The hippocampal damage has been correlated with increased escape latency [72]. The path efficiency also serves as a valid indicator of memory impairment that describes the animals’ ideal route to the platform. The path efficiency also helps to measure the thigmotaxis exhibited by the animals [29]. Additionally, the number of platform entries in the probe trial also serves as a measure of cognitive ability, where a reduced number of entries in the platform region indicates memory decline [73]. Corroborating our results with the parameters accessed in MWM, we believe that animals injected with Aβ 1-42 in our study demonstrated cognitive deficits, thus indicating hippocampal damage. Concurrently, NAT treatment ameliorated the effects of Aβ 1-42-induced cognitive decline and provided neuroprotective effects. It is interesting to note that NAT 30 mg/kg had profound effects on cognition than NAT 50 mg/kg. The MWM measures the characteristics of spatial memory [50]. The improvement in spatial memory performance is mainly regulated by the hippocampal region [74]. Interestingly, when we compared the effect of AChE activity between the two NAT doses (results not shown), we obtained a significant reduction in hippocampal AChE activity by NAT 30 mg/kg as compared to 50 mg/kg (*p*<0.05). This could be suggestive of improved cholinergic signaling by NAT 30 mg/kg in the hippocampus to promote spatial memory functions. The land- and water-based locomotory abilities in rodents differ, reducing the influence of locomotory effects in MWM [75]. However, the differences in the swim speed of animals may correlate to any swimming irregularities [76]. Our analysis of swim speed suggests that the alterations in cognition observed across the treatment groups could be due to spatial impairment and not any swimming abnormalities.

As AD is a progressive neurodegenerative disease involving multiple contributors, neuroinflammation is considered one of the prime contributors to the progression and worsening of the disease. A clinical study in women with AD supported the notion that levels of TNF-α and IL-6 are upregulated in conditions of AD [77]. The administration of Aβ 1-42 oligomers triggered the expression of inflammatory markers like TNF-α and IL-6 [36, 78]. This was consistent with our findings, which showed higher levels of TNF-α and IL-6 in the Aβ 1-42 oligomer-treated animals. Pieces of literature on clinical and animal studies suggested that IL-6 was identified to be elevated in neurodegenerative conditions of CNS involving cognitive decline [54, 79]. Concurrently, Aβ oligomers affected the neurons and the microglia to upregulate TNF-α levels and lead to cognitive decline [80]. Thus, we intended to investigate the role of NAT in neuroinflammatory conditions triggered by Aβ 1-42. Our results demonstrated that NAT posed a dose-dependent reduction in the TNF-α and IL-6 in the hippocampus and frontal cortex, suggesting that NAT could reduce the neuroinflammatory conditions brought on by Aβ. However, the NAT 30 mg/kg failed to reduce the IL-6 levels significantly in our study, which may be related to the high SP levels in animals treated with Aβ 1-42 and NAT 30 mg/kg dose, as depicted in this study. Nevertheless, these findings could support the notion that dose-dependent NAT treatment offered protection against Aβ 1-42-induced neuroinflammation and cognitive decline, evaluated in MWM previously in our study.

Another neuromodulatory marker attaining immense significance in neuroinflammation and cognition is SP. Under stressful conditions, SP levels were upregulated through NK1R activation [81]. The pathophysiology of AD, involving the accumulation of Aβ, is correlated with stressful conditions in the CNS [82]. Therefore, conditions of AD could elevate SP levels. In line with these indications, our study showed similar effects where the SP levels increased in animals treated with Aβ 1-42. This could suggest the activation of NK1R in neuroinflammation and neurodegenerative conditions, as reported elsewhere [83–85]. Previously in our laboratory, *in vivo*, evaluations showed neuroprotective effects of NAT in aluminum chloride-induced spatial memory decline [44], and our *in silico* predictions suggested favorable binding of NAT with NK1R [86]. In this study, NAT showed a dose-dependent reduction in SP levels of the hippocampus and frontal cortex of Aβ 1-42-treated animals. Even though these investigations in our laboratory speculate the NAT-mediated NK1R antagonism, elucidating the exact mechanism is still underway.

Interestingly, SP provoked the upregulation of IL-6 levels [87]. It is worth noting that NAT 30 mg/kg dose failed to reduce the SP levels. Therefore, corroborating the results of IL-6 levels in our study, a link is likely to exist that correlates IL-6 and SP levels in the amyloid beta + NAT 30 mg/kg group mentioned above. Therefore, even though reports suggest neuroprotective actions of SP [88, 89], with caution, we believe that it may promote neurodegenerative actions under specific conditions and the influence of characteristic markers of neuroinflammation and neurodegeneration.

The conditions of AD are often implicated with the upregulation of NFκB signaling. Interestingly, such elevated NFκB signaling selectively triggers an inflammatory action in the dentate gyrus, hampering neuron survival and promoting cognitive decline [90]. Similarly, the levels of NFκB p65 were elevated in the frontal cortex of AD patients [30]. The Aβ activates the NFκB pathway by forming p65 and p50 dimers [91]. The phosphorylation of NFκB p65 at serine 276 (Ser^276^) is shown to mediate the transcriptional activity of p65 to induce neuroinflammation [92]. This could be why, we observed elevated levels of NFκB p65 and p-NFκB p65 in the animals treated with Aβ 1-42 oligomers. Therefore, we evaluated the effect of NAT on the elevated levels of NFκB and p-NFκB-Ser^276^ using western blot analysis. It showed a dose-dependent reduction in the levels of NFκB and p-NFκB compared to the disease group in the hippocampus (Fig. 7a and b) and frontal cortex (Fig. 8a and b). This could suggest the downregulation of the NFκB signaling by NAT, possibly through modulation of p65 phosphorylation at Ser^276^, thus providing a neuroprotective effect.

Along with neuroinflammatory modulators, transcription factors also play a crucial role in AD, including the cAMP response element-binding protein (CREB) [93]. The CREB1 regulates mechanisms that involve memory enhancement [94]. Downregulation of CREB1 signaling upon exposure of hippocampal neurons to Aβ could worsen AD [95, 96]. Concurrently, an interesting study showed that in the prefrontal cortex of AD patients, there was significant downregulation of CREB1 and p-CREB-Ser^133^ [97]. These findings align with our data suggesting that AD pathologies induced by Aβ 1-42 cause a significant reduction in CREB1 and p-CREB-Ser^133^ levels in both the hippocampus and frontal cortex. Our analysis revealed that NAT caused significant upregulation of CREB1 signaling in Aβ 1-42-induced animals. Protein kinase A (PKA) is primarily involved in the phosphorylation of CREB-Ser^133^ [97–100]. These findings influence us to speculate whether NAT could also mediate the upregulation of CREB1 via these pathways.

The AD pathologies are not only characterized by Aβ deposition but also involve the presence of tauopathies. Specific tauopathies related to AD have been identified in the human AD brain [101]. The p-Tau-Ser^396^ forms the highest upregulated form of p-Tau species in the frontal cortex of AD patients associated with Aβ 1-42 [102]. This could explain why we saw upregulation of Tau and p-Tau-Ser^396^ in the hippocampus (Fig. 7e and f) and frontal cortex (Fig. 8e and f) of animals treated with Aβ 1-42. We investigated if NAT was able to reduce Tau and p-Tau-Ser^396^. It significantly downregulated these levels in the hippocampus. Even though it also downregulated Tau levels in the frontal cortex, surprisingly, it failed to elicit any positive response on the p-Tau-Ser^396^ levels.

The cholinergic hypothesis is one of the oldest hypotheses formulated in AD. It states that loss of cognitive function is due to the impairment in the cholinergic system [103]. Since then, efforts have been made to improve cholinergic signaling and cognition in AD using acetylcholinesterase inhibitors [104, 105]. Treatment with Aβ 1-42 provoked upregulation of AChE activity in the hippocampus and frontal cortex in rodents [106]. Similarly, our study observed a marked increase in AChE levels in Aβ 1-42-treated animals. We aimed to ascertain if NAT could reduce the activity of AChE. Interestingly, we observed that NAT treatment showed significant reductions in the AChE levels that could have led to increased Ach levels. Increases in acetylcholine levels are implicated in improved cognition [107, 108]. Therefore, we believe that NAT could positively affect the cholinergic system, which could be the reason, at least in part, for improved cognition, as seen in MWM previously in our study.

It is noteworthy that NAT 50 mg/kg dose showed profound effects on TNF-α, IL6, SP, NFκB, and CREB1 as compared to NAT 30 mg/kg dose. The NFκB directly or indirectly regulates the levels of TNF-α, IL6, SP, and CREB1 [109–111]. The inhibition of NFκB activation can occur through dose-dependent manner of drugs [112, 113]. As NAT 50 mg/kg was shown to effectively reduce NFκB activation in comparison to NAT 30 mg/kg in our results, we speculate that higher NAT concentrations were better capable of preventing transcription of genes that trigger NFKB. Consequently, these events could have a domino effect on the other markers like TNF-α, IL6, SP, and CREB1.

Therefore, corroborating our results, it is evident that markers like NFκB, CREB, Tau, SP, and AChE and inflammatory mediators like TNF-α and IL-6 are inter-connected, especially in the conditions of neurodegenerative conditions like AD. Modulation of TNF-α and IL-6 levels is dependent on the NFκB activity [114, 115]. The NFκB signaling also promotes Tau-mediated toxicity [116]. Similarly, as mentioned before, the IL-6 triggers stimulation of SP levels [87]. A decrease in AChE levels lowered the inflammatory cytokines and promoted neuroprotective actions in AD [117]. The downregulation of NFκB, Tau, SP, and AChE suggests that NAT hampers neuroinflammatory signaling in AD. As a consequence of this, NAT is able to reinforce positive effects on cognitive abilities in neuroinflammation-induced cognitive decline. Concurrently, the upregulation of CREB1 by NAT also elevates cognitive functions and promotes synaptic plasticity, which is affected in AD conditions. Thus, the multi-dimensional effect of NAT on markers like NFκB, CREB, Tau, SP, AChE, TNF-α, and IL-6 could suggest its ability to ameliorate the neuroinflammation-induced cognitive decline in AD.

The HPLC is a robust technique used to determine the brain concentrations of various substrates [118–120]. Amino acids like tryptophan have been identified in rat brains using the HPLC method [121]. We attempted to investigate the levels of NAT in the brains of diseased rats. Surprisingly, our HPLC bioanalytical method was not sensitive enough to detect NAT in rat brains. We suspect that the levels of NAT may lie below the LLOQ of our HPLC bioanalytical method. LLOQ is the lowest level of analyte concentrations that can be detected with 20% precision [122]. Therefore, NAT may be present in the brain in concentrations lesser than 1 μg/ml. To support these findings, we performed simulations using GastroPlus. The GastroPlus can simulate drug absorption parameters through multiple administration routes and predict drug absorption, distribution, metabolism, and excretion in humans and animals [123]. It has been employed in multiple studies to perform *in vivo* pharmacokinetic predictions [124–126]. The Cmax of i.v bolus and oral suspension of NAT showed lower levels. If these predicted values are extrapolated to the brain concentrations, then NAT could be present in concentrations less than that in the plasma. As a result, NAT remained undetected in the brain samples using the HPLC mode of analysis. Another study reported that tryptophan, an amino acid having structural similarity to NAT, showed lower than normal levels in the plasma and brain of obese patients after 24 h [127]. Further, the fact that NAT remained undetected in brain samples could be a possible rationale for the existence of few literature reports on the quantification of NAT in the brain using the HPLC method. Therefore, advanced sensitive techniques like LC-MS and LC-MS-MS could be an alternative method to quantify NAT at the nanogram level, specifically with the biological matrixes.

## Conclusion

The neurodegeneration associated with AD involves multi-spoked pathological complications, and combating all of which is still a challenge, yet every researcher’s dream. The magnitude of AD makes it necessary to develop efficient therapies with a broader range of actions. Our study demonstrates that NAT withholds the potential to exert neuroprotective actions in AD conditions by ameliorating the cognitive decline and neuroinflammation induced by Aβ 1-42. Even though this encourages us to have optimistic speculations about its possible mechanism of action, further studies are warranted to uncover NAT’s underlying signaling that would eventually help target AD even better.

## Data Availability

The data that support the findings of this study are available from the corresponding author, upon reasonable request.
